# Baseline characteristics and response to evinacumab in females and males with homozygous familial hypercholesterolemia in the ELIPSE OLE study^☆^^☆☆^

**DOI:** 10.1016/j.ajpc.2025.101395

**Published:** 2026-01-19

**Authors:** Diane Brisson, Albert Wiegman, Alpana Waldron, Pinay Kainth, Frederick J Raal, Daniel Gaudet

**Affiliations:** a Université de Montréal and ECOGENE-21, Chicoutimi, Québec, Canada; b Department of Pediatrics, Amsterdam University Medical Centers, The Netherlands; c Regeneron Pharmaceuticals, Inc., Tarrytown, NY, USA; d Ultragenyx Pharmaceutical Inc., Novato, CA, USA; e Faculty of Health Sciences, University of the Witwatersrand, Johannesburg, South Africa

**Keywords:** Homozygous familial hypercholesterolemia, LDL-cholesterol, Sex, Treatment

## Abstract

**Aim:**

Evinacumab is an ANGPTL-3 inhibitor developed for the treatment of homozygous familial hypercholesterolemia (HoFH), a rare condition characterized by extremely elevated LDL-cholesterol (LDL-C) levels and premature atherosclerotic cardiovascular disease. Despite important sex-related disparities in lipid metabolism, females are still underrepresented in trials, limiting the generalizability of results. With 116 patients, of which 49 % were females, the ELIPSE-OLE study has the largest cohort of HoFH females involved in a clinical trial. The aim of this analysis was to compare response to evinacumab in females and males in the ELIPSE OLE study.

**Methods:**

This study is a post hoc exploratory analysis of data on 57 females and 59 males, aged ≥12 years and on stable background lipid-lowering therapy (LLT) from ELIPSE OLE. Patients received evinacumab 15 mg/kg intravenously every 4 weeks. The primary efficacy endpoint was the reduction in LDL-C concentration from baseline to week-24.

**Results:**

Baseline LDL-C [mean (SD)] tended to be higher in females aged 18 to <50 than males: 8.4(5.4) vs 6.4(3.5) mmol/L. Following treatment with evinacumab, the percent decrease in LDL-C reached at week-24 was 44 % in the overall cohort and was sustained over time. Evinacumab significantly decreased LDL-C in both sexes, regardless of age and background LLT. There was a trend, although not significant, toward higher relative precent decrease of LDL-C among females.

**Conclusion:**

In a study where half of the participants were females, evinacumab led to substantial LDL-C reduction in HoFH patients of both sexes, regardless of genotype or background LLT.

## Graphical abstract

**Figure:**
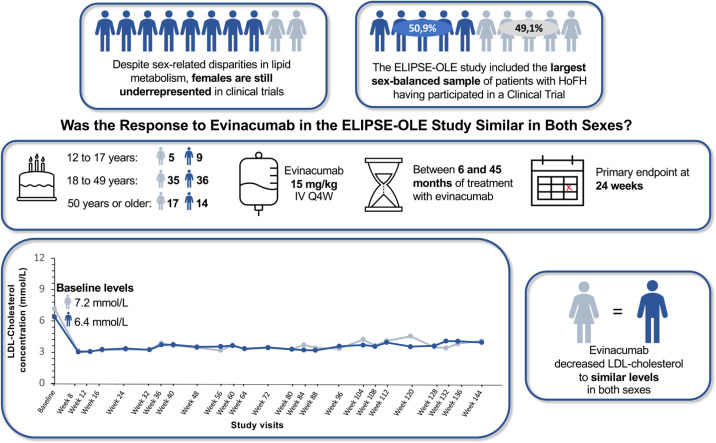

ELIPSE-OLE, Open-Label Extension to Evinacumab LIPid StudiEs in Patients With HoFH; IV, Intravenous, HoFH, Homozygous familial hypercholesterolemia, LDL, Low-density lipoproteins; Q4W, Every 4 weeks

## 1 Introduction

Homozygous familial hypercholesterolemia (HoFH) is a rare condition characterized by extremely elevated low-density lipoprotein-cholesterol (LDL-C) levels, which typically exceed 500 mg/dL (13 mmol/L), and premature atherosclerotic cardiovascular disease (ASCVD) [1,2]. Its prevalence ranges between 1:400,000 to 1:160,000 but may be even higher in some founder populations [3,4]. HoFH is mainly caused by the presence of bi-allelic pathogenic variants in the LDL receptor (*LDLR*), apolipoprotein B (*APOB*) or proprotein convertase subtilisin/kexin type 9 (*PCSK9*) genes, resulting in a complete or partial impairment of LDL-C clearance from plasma via LDLR [1,2,5].

Clinical guidelines for HoFH patients recommend reduction of LDL-C levels to <1.4 mmol/L and <1.8 mmol/L for adults with and without ASCVD-risk factors or established ASCVD, respectively. For children and adolescents, the clinical target is LDL-C level <3 mmol/L, if lipid-lowering therapy (LLT) is initiated before the age of 18 years and imaging assessment does not indicate the presence of ASCVD, or lower if ASCVD is established [4]. However, these LDL-C levels are difficult to reach in HoFH, even when combining several treatment strategies. LDLR activity is an important determinant of response to LLTs that influence the capacity for achieving clinical targets. Patients with biallelic LDLR null/null variants generally respond poorly to statins and ezetimibe and very poorly, or not at all, to PCSK9 inhibitors, whose predominant mechanism of action is via LDLR upregulation [4]. Lomitapide and lipoprotein apheresis are LDLR-independent treatment options for HoFH patients. Lomitapide is not yet approved for patients aged <18 years and is associated with gastrointestinal and hepatic adverse events [6]. Lipoprotein apheresis is an invasive, tiresome and time-consuming treatment not easily accessible to all patients [4]. Research in recent years have therefore focused on the development of new treatments with LDLR independent modes of action.

Evinacumab is a recombinant human monoclonal antibody (mAb) inhibiting angiopoietin-like protein 3 (ANGPTL3), a circulating protein synthesized in the liver with pleiotropic modulating effects on lipid-lipoprotein metabolism [7,8]. ANGPTL3 inhibition leads to enhanced lipoprotein clearance upstream of LDL and independently of the LDLR function [8]. Evinacumab was shown to decreased LDL-C concentrations by ∼50 % in HoFH patients aged ≥12 years after 24 weeks with concomitant maximally tolerated LLT, irrespective of apheresis status. It was approved as an add-on treatment to other LLTs for HoFH patients, based on results of the pivotal phase 3 ELIPSE-HoFH clinical trial (Evinacumab LIPid StudiEs in Patients With HoFH) [9].

Important sex-related disparities are well-known to exist in lipid-lipoprotein metabolism. Several studies and real-world data also demonstrate significant differences in LDL-C target attainment, LLT-related side effects and adherence to LLT between males and females with dyslipidemia [10,11]. Unfortunately, despite this, females are still underrepresented in LLT clinical trials, limiting the generalizability of safety and efficacy outcomes [12]. Issues associated with the underrepresentation of females are even more important for rare dyslipidemias, like HoFH, for which the very small sample sizes greatly limit the ability to perform post hoc analyses among sex-based subgroups.

With 118 screened and 116 enrolled patients, of which 49 % were females, the ELIPSE-OLE study (Open-Label Extension to Evinacumab LIPid StudiEs in Patients With HoFH) is the largest cohort of HoFH patients, and of HoFH females, involved in a clinical study to date [13,14]. It offers the possibility to perform baseline and longitudinal sex-based subgroup analyses to better document HoFH expression or factors that influence response to evinacumab over time. The aim of this analysis was to compare response to evinacumab in females and males in the ELIPSE-OLE study.

## 2 Methods

### 2.1 Study design and patients

This study is a post hoc exploratory analysis of data obtained in the ELIPSE OLE study. The ELIPSE OLE study was an open-label phase 3 extension study conducted in 38 sites across 12 countries to assess the long-term safety and efficacy of evinacumab in HoFH patients aged ≥12 years (NCT03409744). Details on study design and eligibility criteria have been previously published [13]. Briefly,116 patients (57 females and 59 males) with a genetic or clinical diagnosis of HoFH were enrolled. All patients had to be on stable background LLT at study screening. They were either evinacumab naïve (*n* = 46) or had previously received evinacumab during their participation in the phase 2 proof-of-concept study (NCT02265952) or the phase 3 ELIPSE-HoFH study (NCT03399786) (*n* = 70) [8,9]. Patients received evinacumab 15 mg/kg intravenously at baseline (Day 1) and every 4 weeks (Q4W) for up to 192 weeks. Patients gave their informed consent to participate in this study. This study was conducted in accordance with the Declaration of Helsinki and was approved by the institutional review board and/or ethics committee at each site.

### 2.2 Endpoints

The primary endpoint of the ELIPSE OLE study was the long-term safety and tolerability of evinacumab. Its primary efficacy endpoint was the relative (%) and absolute change in LDL-C concentrations from baseline to week 24. The present analysis compared baseline characteristics, LDL-C response to evinacumab as well as safety and tolerability of evinacumab between females and males in the whole sample and by age subgroups in the ELIPSE OLE trial.

### 2.3 Data collection

Demographics, anthropometrics, risk factors, cardiovascular history, and concomitant LLT were collected at baseline for each patient. Baseline was defined as the last value obtained before the first dose of evinacumab for patients who had previously received evinacumab in the phase 2 study or who were evinacumab naïve [8]. For patients who had previously received evinacumab in the Phase 3 study, baseline was defined as the last obtained value before the first dose of double-blind evinacumab in the Phase 3 study [9]. Blood samples were collected before evinacumab administration and, in those receiving lipoprotein apheresis just prior to apheresis, for measurement of lipid profile by the central laboratory of the ELIPSE HoFH—OLE trial.

### 2.4 Statistical analysis

This was not a prespecified analysis and no sample size calculation was performed. Efficacy data were analyzed for all patients who received at least one dose or partial dose of evinacumab 15 mg/kg in the ELIPSE HoFH—OLE trial. Patients’ baseline characteristics and relative ( %) and absolute changes from baseline in LDL-C were summarized using descriptive statistics. Inferential statistical analysis was performed using immediate commands that allow to run tests with summary statistics. Categorical variables were compared using the Pearson Chi^2^ statistic or Fisher’s exact tests, and group differences for continuous variables were compared with unpaired two-tailed Student-t tests or with the Welch’s *t*-test. Missing data were not imputed. All p-values are nominal without any adjustments for multiple comparisons or other factors. Statistical analyses were performed using Stata 13.1 (College Station, TX, USA).

## 3 Results

As shown in Table 1, males and females were comparable for all variables except for HDL-C concentration, which was higher among females (*p* < 0.0001). They were also comparable in terms of HoFH diagnostic criteria and proportion of null-null *LDLR* variant carriers when separated according to age (Table 2). Males aged ≥50 years have higher body mass index (BMI) (*p* = 0.03) and those aged <18 years tended to be older than females (*p* = 0.07). Background LLT intensity was similar, except for lomitapide, which was more often used in males aged 18 to <50 years (*p* = 0.01), and lipoprotein apheresis, which was used more frequently among females ≥50 years (*p* = 0.0009). Similar proportion of males and females reported previous tolerability issues that led to down titration or change to different statin (data not shown). Overall, females of the current study have higher HDL-C concentrations than males. There was no difference in other lipid parameters at baseline between males and females aged <18 years or ≥50 years. Baseline LDL-C and non-HDL-C [mean (SD)] tended to be higher in females aged 18 to <50 than in males: 8.4 (5.4) vs 6.4 (3.5) mmol/L (*p* = 0.07) and 9.0 (5.5) vs 6.9 (3.5) mmol/L (*p* = 0.06) (Table 2). Important intra-group variation may explain why such large differences did not reach significance levels.

**Table 1:** Patients’ baseline characteristics.

		**Females** ***n*=57**	**Males** ***n*=59**	***P-*value**
Age, years		40.7 (16.4)	37.1 (15.4)	NS
BMI, kg/m^2^		24.7 (4.9)	26.5 (6.5)	NS
Diagnosis, n ( %)*				NS
	Genotyping	45 (79)	40 (68)	
	Clinical	12 (21)	19 (32)	
Null/Null *LDLR* variant, n ( %)		9 (15.8)	8 (13.6)	NS
Baseline LLT, n ( %)				
	Statin	56 (98)	54 (92)	NS
	High int. statin	55 (96)	54 (92)	NS
	Ezetimibe	49 (86)	49 (83)	NS
	PCSK9 inhibitor	40 (70)	35 (59)	NS
	Apheresis	22 (39)	21 (36)	NS
	Lomitapide	8 (14)	15 (25)	NS
LDL-C, mmol/L		7.2 (4.8)	6.4 (3.4)	NS
Apo B, g/L		1.8 (1.0)	1.7 (0.7)	NS
HDL-C, mmol/L		1.3 (0.4)	0.9 (0.2)	<0.0001
Non-HDL-C, mmol/L		7.7 (4.9)	6.9 (3.4)	NS

Data are mean (SD) unless otherwise specified. NS: *P* value >0.1

*At study enrolment.

Apheresis, Lipoprotein apheresis; Apo B, Apolipoprotein B; BMI, Body mass index; HDL-C, High-density lipoprotein-cholesterol; High int. Statin, High intensity statin; LDL-C, Low-density lipoprotein-cholesterol; LLT, lipid lowering therapy; NS, Non-significant; SD, Standard deviation.

**Table 2:** Patients’ baseline characteristics according to age and sex.

		**Aged 12 to <18 years**			**Aged 18 to <50 years**			**Aged ≥50 years**		
		**Females** ***n*=5**	**Males** ***n*=9**	***P v*alue**	**Femalesn=35**	**Males** ***n*=36**	***P* value**	**Femalesn=17**	**Males** ***n*=14**	***P* value**
Age, years		13.2 (1.6)	15.0 (1.7)	0.07	35.2 (9.0)	34.3 (7.9)	NS	60.0 (8.4)	58.4 (5.8)	NS
BMI, kg/m^2^		19.5 (2.8)	20.5 (3.3)	NS	25.3 (4.3)	26.6 (6.2)	NS	25.1 (5.8)	30.1 (6.5)	0.03
Diagnosis, n ( %)*				NS			NS			NS
	Genotyping	5 (100)	6 (67)		29 (83)	25 (69)		11 (65)	9 (64)	
	Clinical	0	3 (33)		6 (17)	11 (31)		6 (35)	5 (36)	
Null/Null *LDLR* variant, n ( %)		2 (40)	2 (25)^1^	NS	6 (17.1)	6 (17)	NS	1 (6)	0	NS
Baseline LLT, n ( %)										
	Statin	5 (100)	9 (100)	NS	34 (97)	32 (89)	NS	17 (100)	13 (93)	NS
	High int. statin	5 (100)	9 (100)	NS	34 (97)	32 (89)	NS	16 (94)	13 (93)	NS
	Ezetimibe	5 (100)	9 (100)	NS	31 (89)	30 (83)	NS	13 (77)	10 (71)	NS
	PCSK9 inhibitor	3 (60)	4 (44)	NS	27 (77)	21 (58)	0.09	10 (59)	10 (71)	NS
	Apheresis	2 (40)	7 (78)	NS	11 (31)	13 (36)	NS	9 (53)	1 (7)	0.009
	Lomitapide	0	1 (11)	NS	3 (9)	12 (33)	0.01	5 (29)	2 (14)	NS
LDL-C, mmol/L		7.6 (3.4)	7.9 (2.3)	NS	8.4 (5.4)	6.4 (3.5)	0.07	4.4 (2.2)	5.2 (3.4)	NS
Apo B, g/L		1.9 (0.7)	1.9 (0.5)	NS	2.0 (1.1)	1.7 (0.7)	NS	1.2 (0.5)	1.5 (0.6)	NS
HDL-C, mmol/L		1.2 (0.4)	1.0 (0.2)	NS	1.2 (0.4)	0.9 (0.2)	0.0006	1.4 (0.5)	1.0 (0.3)	0.009
Non-HDL-C, mmol/L		8.2 (3.2)	8.3 (2.4)	NS	9.0 (5.5)	6.9 (3.6)	0.06	4.9 (2.3)	6.0 (3.3)	NS

Data are mean (SD) unless otherwise specified. NS: *P* value >0.1.

*At study enrolment.

Apheresis, Lipoprotein apheresis; Apo B, Apolipoprotein B; BMI, Body mass index; HDL-C, High-density lipoprotein-cholesterol; High int. Statin, High intensity statin; LDL-C, Low-density lipoprotein-cholesterol; LLT, lipid lowering therapy; NS, Non-significant; SD, Standard deviation.

**Fig. 1:**
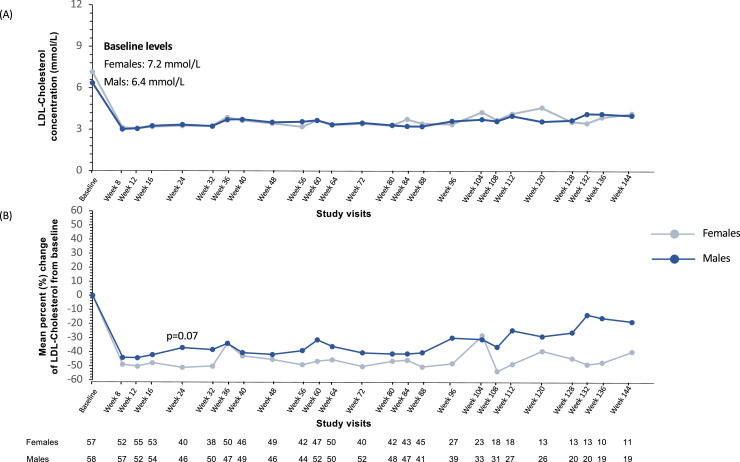
Mean plasma LDL-cholesterol (A) and relative ( %) change (B) in LDL-cholesterol concentrations from baseline to week 144 by sex. Relative change was calculated from LDL-cholesterol concentration at baseline, pre-evinacumab treatment. LDL: Low-density lipoproteins.

**Fig. 2:**
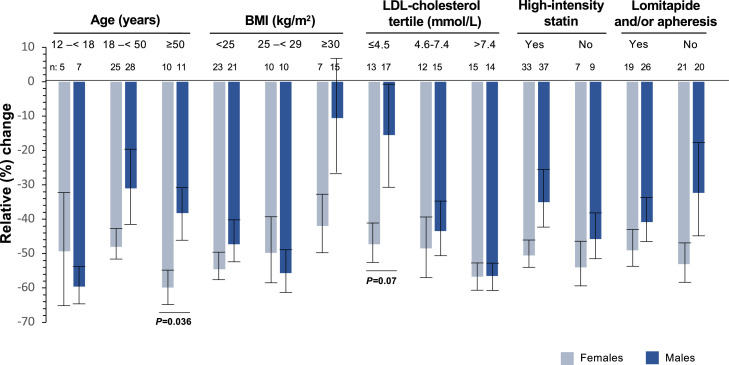
Mean ± SE relative ( %) change in LDL-cholesterol from baseline to Week 24 by sex according to patients’ characteristics at baseline. Relative change was calculated from LDL-cholesterol concentration at baseline, pre-evinacumab treatment. BMI, Body mass index; LDL*,* Low-density lipoprotein; SE, Standard error.

Evinacumab treatment substantially and incrementally decreased LDL-C concentration and similar levels were reached by both males and females (Fig. 1A). As baseline LDL-C tended to be higher among females, relative ( %) decrease tended to be higher among the latter (Fig. 1B). It was among patients aged ≥50 years that the most significant difference between males and females in relative decrease of LDL-C at week 24 was observed (*p* = 0.036). There was no significant difference between males and females in decreases of LDL-C at week 24, irrespective of the baseline BMI, LDL-C levels, use of high-intensity statin or concomitant use of LDLR-independent treatment (lomitapide and/or apheresis). However, decreases in baseline LDL-C at week 24 tended to be higher in females than in males in patients with baseline LDL-*C* ≤ 4.5 mmol/L (−47 % vs. −15 % [−1.21 (0.7) mmol/L vs. −0.8 (1.4) mmol/L]) (Fig. 2).

**Fig. 3:**
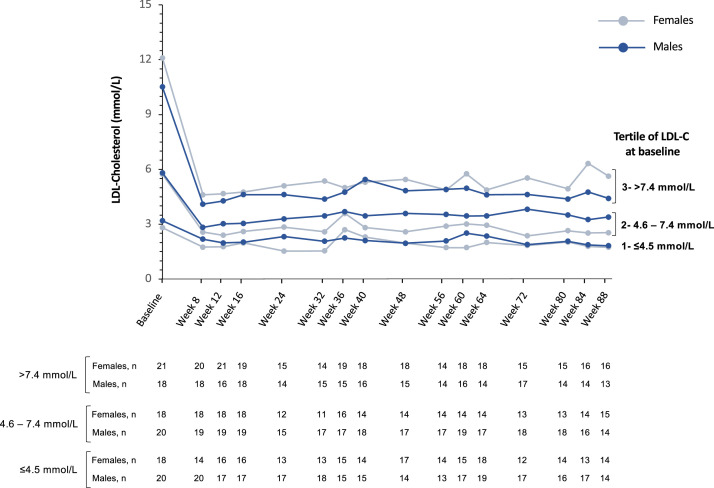
Mean LDL-cholesterol concentrations over time by sex, according to tertile of LDL-cholesterol at baseline. Relative change was calculated from LDL-cholesterol concentration at baseline, pre-evinacumab treatment. LDL*,* Low-density lipoprotein.

**Fig. 4:**
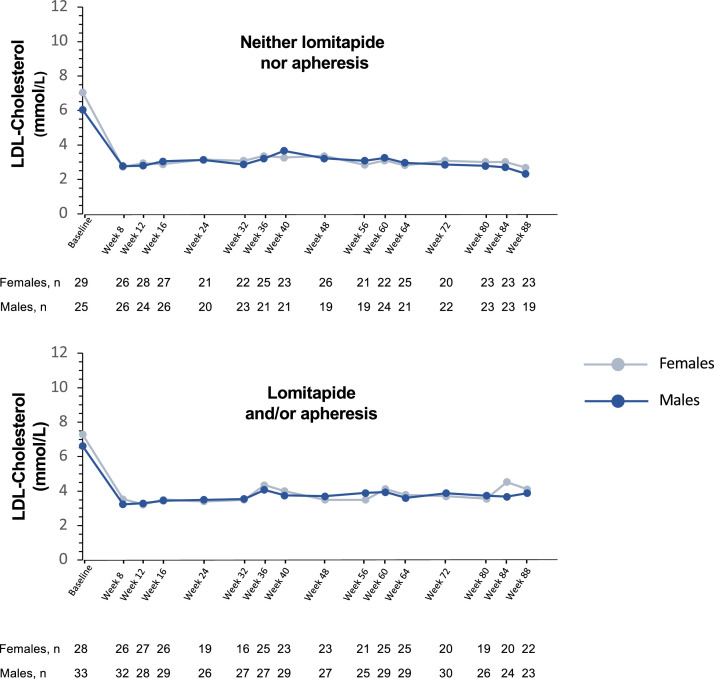
Mean LDL-cholesterol concentrations over time by sex, according to the use of LDLR-independent LLT at baseline. Relative change was calculated from LDL-cholesterol concentration at baseline, pre-evinacumab treatment. LDL, Low-density lipoprotein; LDLR*,* Low-density lipoprotein receptor; LLT, Lipid-lowering therapy.

Reductions in LDL-C concentrations were sustained over time regardless of tertile of LDL-C levels at baseline and of the concomitant use of LDLR-independent treatment. Although there were some differences between males and females in absolute and relative decreases of LDL-C, the only significant difference was at week 72 among patients in tertile 2 (4.6 to 7.4 mmol/L) of baseline LDL-C concentration (*p* = 0.004) (Fig. 3). Similar levels of LDL-C were reached by both males and females among patients whether they received lomitapide and/or lipoprotein apheresis, even if females tended to have higher baseline LDL-C levels in both subgroups (Fig. 4).

Overall, there was no significant sex-specific differences in treatment emergent adverse events (TEAEs), except for gastroesophageal reflux disease, which was reported only by females, and a higher prevalence of urinary tract infection among females (Table 3).

**Table 3:** Summary of treatment-emergent adverse events according to sex.

**n ( %) of patients**	**Females** ***n*=57**	**Males** ***n*=59**
Any TEAE	48 (84.2)	45 (76.3)
Serious TEAEs	16 (28.1)	11 (18.6)
TEAEs resulting in treatment discontinuation	3 (5.3)	0
TEAE resulting in death	1 (1.8)	1 (1.7)
TEAEs occurring in ≥5 % of patients by preferred term		
Anemia	3 (5.3)	1 (1.7)
Palpitations	3 (5.3)	0
Hypothyroidism	3 (5.3)	1 (1.7)
Nausea	4 (7.0)	10 (16.9)
Diarrhea	3 (5.3)	7 (11.9)
Abdominal pain	3 (5.3)	4 (6.8)
Toothache	3 (5.3)	4 (6.8)
Vomiting	2 (3.5)	5 (8.5)
Gastroesophageal reflux disease	**6 (10.5)**	**0**
Influenza like illness	5 (8.8)	11 (18.6)
Pyrexia	6 (10.5)	4 (6.8)
Chest pain	3 (5.3)	1 (1.7)
Oedema peripheral	3 (5.3)	1 (1.7)
Non-alcoholic steatohepatitis	0	3 (5.1)
Immunization reaction	3 (5.3)	2 (3.4)
Seasonal allergy	3 (5.3)	0
Nasopharyngitis	10 (17.5)	13 (22.0)
COVID-19	11 (19.3)	8 (13.6)
Gastroenteritis	4 (7.0)	6 (10.2)
Urinary tract infection	**8 (14.0)**	**2 (3.4)**
Upper respiratory tract infection	3 (5.3)	5 (8.5)
Rhinitis	1 (1.8)	3 (5.1)
Contusion	4 (7.0)	2 (3.4)
Apheresis related complication	3 (5.3)	0
Alanine aminotransferase increased	1 (1.8)	3 (5.1)
Aspartate aminotransferase increased	1 (1.8)	3 (5.1)
Blood creatine phosphokinase increased	1 (1.8)	3 (5.1)
Arthralgia	6 (10.5)	9 (15.3)
Back pain	5 (8.8)	9 (15.3)
Pain in extremity	5 (8.8)	3 (5.1)
Myalgia	2 (3.5)	5 (8.5)
Muscle spasms	3 (5.3)	1 (1.7)
Tendonitis	0	3 (5.1)
Headache	9 (15.8)	10 (16.9)
Dizziness	5 (8.8)	2 (3.4)
Pregnancy	3 (5.3)	0
Cough	5 (8.8)	7 (11.9)
Oropharyngeal pain	3 (5.3)	3 (5.1)
Rhinorrhea	3 (5.3)	0
Sleep apnea syndrome	0	3 (5.1)
Dermatitis contact	3 (5.3)	0

TEAE; Treatment-emergent adverse events.

## 4 Discussion

This analysis showed that evinacumab substantially and incrementally decreased LDL-C and brought plasma concentrations to similar levels among males and females, even though females, especially those aged 18 to <50 years, tended to have higher baseline LDL-C levels.

Globally, males and females were comparable in terms of HoFH diagnostic criteria, proportion of null-null *LDLR* variant carriers and other variables in all age groups. Not surprisingly – and similar to what has been documented in the general population and, more recently, among patients with HoFH [15,16] – females aged ≥18 years tend to have higher plasma HDL-C than males. The trend for baseline LDL-C and non-HDL-C to be higher among females aged 18 to <50 years was unexpected. Higher treated total and LDL-C concentrations were indeed previously observed among females with heterozygous familial hypercholesterolemia (HeFH), as compared to HeFH males, despite similar treatment [17,19], but no sex difference was observed in treated LDL-C among HoFH patients in the few recent studies [16,20]. However, in these studies, comparisons were not performed according to patient’s age groups, which may explain the apparent divergence with our results. In the current study, treated LDL-C and non-HDL-C measured at baseline were indeed not significantly different between both sexes when comparisons were performed in the whole sample.

Several studies, including a cross-sectional study from the European Atherosclerosis Society Familial Hypercholesterolemia Studies Collaboration (FHSC) global registry, the largest registry of FH patients worldwide, reported that HeFH females are less likely to be on LLT, receive less potent LLT and use combination therapies less often than males [18,21,22]. They also reported treatment-related side-effects more frequently, thus preventing LLT up-titration to optimal intensities and reducing treatment adherence, which can partly explain higher treated LDL-C concentrations among them [21]. Such differences between males and females in LLT type and intensity were not observed in HoFH, which can be explained by the greater severity of the disease and the fact that most patients are followed in specialized lipid clinics [16,20]. In the current study, LLT intensity at baseline was comparable in both sexes and in all age groups. Lomitapide was significantly more often used among males aged 18 to <50 years than females, which could be the likely reason for the sex-related disparities observed in treated baseline LDL-C and non-HDL-C in this age group. However, even if similar proportion of males and females reported previous statin-related tolerability issues, breaches in treatment adherence for other LLTs cannot completely be excluded. As severity of the disease and specialized care contribute to treatment adherence [23], this variable may be of lesser importance among HoFH patients.

As noted in the previous analyses, LDL-C tended to be higher among females at baseline, with similar LDL-C concentrations reached in both males and females following evinacumab treatment, resulting in a trend toward higher relative percent decreases in females [13,14]. However, reductions in LDL-C concentrations were sustained over time regardless of tertile of LDL-C levels at baseline. Interestingly, similar concentrations of LDL-C were reached in both males and females, whether they received LDLR-independent treatment (lomitapide and/or apheresis) or not, and even although females tended to have higher baseline LDL-C levels in both subgroups. Equivalent results were observed with lomitapide treatment in the Pan-European Lomitapide Study [24], which also showed a trend toward a greater reduction in LDL-C in HoFH female compared with males after 6 months of lomitapide treatment. In their subanalysis, males and females had similar baseline LDL-C levels [24], unlike what was observed in our current study. Sex-related disparities are known to influence lipid kinetics, which could explain differences between males and females in response to LLT. Some interesting hypotheses could also be drawn, based on what is known about the metabolic pathway for evinacumab and ANGPTL3. Although no direct effect of estrogens on ANGPTL3 expression or activity has been documented, their potential modulator effect could take place upstream and/or downstream in the metabolic pathway. Sex-related differences in drug absorption, distribution and metabolism are other potential modulators of treatment effectiveness [25]. Evinacumab is a human monoclonal immunoglobulin (Ig) G4: it is therefore expected to be degraded similar to endogenous IgG [26]. Studies have shown significant sex-based variability in mAb clearance and efficacy and that estrogens can be significant modulators of IgG metabolism [27,28]. One can therefore hypothesize that this could potentially impact the metabolism of evinacumab.

Evinacumab was generally well tolerated in both males and females, adult and adolescent, as previously reported [13,14]. The tolerability profile of evinacumab was quite similar in males and females, except for a higher prevalence of gastrooesophageal reflux disease and urinary tract infections among females. Heartburn and non-erosive reflux disease as well as urinary track infections being more common in women [29,30], this observation was not surprising.

The main strength of the ELIPSE OLE study is the inclusion of a large sex-balanced multiethnic cohort of well-characterized patients with HoFH. As far as we are aware, it is the largest cohort of HoFH (and of HoFH females) reported in a clinical study. ELIPSE OLE baseline and longitudinal data offer potential to support global initiatives and facilitating equitable access to innovation for affected HoFH patients without discrimination. The post-hoc exploratory nature of these analyses performed with data obtained from a study with a non-randomized open-label design is however associated with various methodological limitations, including the increased risk of type I errors (false positives). In addition, although its sample size was large for an ultra-rare disease, a limitation of the study is that the cohort was relatively small, which limited the statistical power and reduced the possibilities of sub-analyses. Another limitation was access to grouped data for some of the parameters which limited the number and type of statistical analyses that could be performed. It was therefore not possible to analyze group differences for the proportion of patients achieving LDL-C clinical targets or to perform multivariate analyses and to adjust, as instance, for baseline LDL-C. These analyses could have improved data interpretation.

Results of the current study are obviously insufficient on their own to change therapeutic decision-making. However, even if the observed differences are statistically modest, they provide additional evidence that achieving equity in access to adapted healthcare requires addressing the imbalance in the representation of females versus males in clinical trials, especially for rare disorders. Multiplication of global initiatives, including conducting sex-balanced clinical trials and post-hoc analyses designed to document sex-specific drugs effects, are crucial to ensure equitable access to innovation.

## 5 Conclusion

In a study where half of the participants were females, evinacumab substantially and incrementally decreased LDL-C and brought plasma concentrations to similar levels among males and females, regardless of the genotype or background LLT (Graphical abstract).

## Funding

This study was supported by Regeneron Pharmaceuticals, Inc.

## Data availability

Qualified researchers may request access to study documents (including the clinical study report, study protocol with any amendments, blank case report form, and statistical analysis plan) that support the methods and findings reported in this manuscript. Individual anonymized participant data will be considered for sharing once the product and indication has been approved by major health authorities (eg- FDA, EMA, PMDA, etc.), if there is legal authority to share the data and there is not a reasonable likelihood of participant re-identification. Submit requests to https://vivli.org/.

## Conflicting of interest

D Brisson reports no conflict of interest.

A Wiegman reports research grants, honoraria, and/or consulting fees for professional input and/or delivered lectures from Amgen, Chiesi, Esperion, Merck, Novartis, Regeneron, Sanofi, Silence Therapeutics and Ultragenyx. All are received by the Amsterdam University Medical Centers.

A Waldron is an employee and stock holder of Regeneron Pharmaceuticals, Inc. and a stock holder of Bristol Myers Squibb

P Kainth is an employee and stock holder of Ultragenyx Pharmaceutical Inc.

FJ Raal has received research grants, honoraria, and/or consulting fees for professional input and/or delivered lectures from Novartis, Regeneron, Ultragenyx, Chiesi, Silence Therapeutics, Verve Therapeutics and LIB Therapeutics.

D Gaudet reports research grants, honoraria, and/or consulting fees for professional input and/or delivered lectures from 89Bio, Alnylam, Amgen, Applied Therapeutics, Arrowhead, Astra Zeneca, Boehringer-Ingelheim, Chiesi (Amryt), CRISPR Therapeutics, Dalcor Pharma, Eli Lilly, Esperion, Fenix Group, Flagshippionnering, Inversago, Ionis, Kowa, Merck, New Amsterdam, NovoNordisk, Regeneron, Rona Therapeutics, Saliogen, The Medicine Company, Ultragenyx, Verve Therapeutics. Research grant payments are received by ECOGENE-21, an academic non-profit research organization.

## CRediT authorship contribution statement

**Diane Brisson:** Writing – original draft, Formal analysis, Conceptualization. **Albert Wiegman:** Writing – review & editing, Investigation. **Alpana Waldron:** Writing – review & editing, Data curation. **Pinay Kainth:** Writing – review & editing, Data curation. **Frederick J Raal:** Writing – review & editing, Investigation. **Daniel Gaudet:** Writing – review & editing, Investigation, Conceptualization.

## Declaration of competing interest

The authors declare the following financial interests/personal relationships which may be considered as potential competing interests: Daniel Gaudet reports financial support was provided by Regeneron Pharmaceuticals Inc. Albert Wiegman reports a relationship with Amgen, Chiesi, Esperion, Merck, Novartis, Regeneron, Sanofi, Silence Therapeutics and Ultragenyx. All are received by the Amsterdam University Medical Centers that includes: board membership, consulting or advisory, funding grants, and speaking and lecture fees. Alpana Waldron reports a relationship with Regeneron Pharmaceuticals Inc that includes: employment and equity or stocks. Pinay Kainth reports a relationship with Ultragenyx Pharmaceutical Inc that includes: employment and equity or stocks. Frederick J Raal reports a relationship with Novartis, Regeneron, Ultragenyx, Chiesi, Silence Therapeutics, Verve Therapeutics and LIB Therapeutics that includes: board membership, consulting or advisory, funding grants, and speaking and lecture fees. Alpana Waldron reports a relationship with Bristol Myers Squibb that includes: equity or stocks. Daniel Gaudet reports a relationship with 89Bio, Alnylam, Amgen, Applied Therapeutics, Arrowhead, Astra Zeneca, Boehringer-Ingelheim, Chiesi (Amryt), CRISPR Therapeutics, Dalcor Pharma, Eli Lilly, Esperion, Fenix Group, Flagshippionnering, that includes: board membership, consulting or advisory, funding grants, and speaking and lecture fees. Daniel Gaudet reports a relationship with Inversago, Ionis, Kowa, Merck, New Amsterdam, NovoNordisk, Regeneron, Rona Therapeutics, Saliogen, The Medicine Company, Ultragenyx, Verve Therapeutics that includes: board membership, consulting or advisory, funding grants, and speaking and lecture fees. If there are other authors, they declare that they have no known competing financial interests or personal relationships that could have appeared to influence the work reported in this paper.
